# Advances in MIMO Antenna Design for 5G: A Comprehensive Review

**DOI:** 10.3390/s23146329

**Published:** 2023-07-12

**Authors:** Tej Raj, Ranjan Mishra, Pradeep Kumar, Ankush Kapoor

**Affiliations:** 1 Electrical Cluster, SoE, UPES, Dehradun 248007, India; tej.107543@stu.upes.ac.in (T.R.); rmishra@ddn.upes.ac.in (R.M.); 2 Discipline of Electrical, Electronic and Computer Engineering, University of KwaZulu-Natal, Durban 4041, South Africa; 3 Department of Electronics and Communication Engineering, Jawaharlal Nehru Government Engineering College, Sundernagar 175018, India; ankush.8818@hp.gov.in

**Keywords:** 5G, B5G (beyond 5G), wideband MIMO, multiband MIMO, FR-I, FR-II, mmWave

## Abstract

Multiple-input multiple-output (MIMO) technology has emerged as a highly promising solution for wireless communication, offering an opportunity to overcome the limitations of traffic capacity in high-speed broadband wireless network access. By utilizing multiple antennas at both the transmitting and receiving ends, the MIMO system enhances the efficiency and performance of wireless communication systems. This manuscript specifies a comprehensive review of MIMO antenna design approaches for fifth generation (5G) and beyond. With an introductory glimpse of cellular generation and the frequency spectrum for 5G, profound key enabling technologies for 5G mobile communication are presented. A detailed analysis of MIMO performance parameters in terms of envelope correlation coefficient (ECC), total active reflection coefficient (TARC), mean effective gain (MEG), and isolation is presented along with the advantages of MIMO technology over conventional SISO systems. MIMO is characterized and the performance is compared based on wideband/ultra-wideband, multiband/reconfigurable, circular polarized wideband/circular polarized ultra-wideband/circular polarized multiband, and reconfigurable categories. The design approaches of MIMO antennas for various 5G bands are discussed. It is subsequently enriched with the detailed studies of wideband (WB)/ultra-wideband (UWB), multiband, and circular polarized MIMO antennas with different design techniques. A good MIMO antenna system should be well decoupled among different ports to enhance its performance, and hence isolation among different ports is a crucial factor in designing high-performance MIMO antennas. A summary of design approaches with improved isolation is presented. The manuscript summarizes the various MIMO antenna design aspects for NR FR-1 (new radio frequency range) and NR FR-2, which will benefit researchers in the field of 5G and forthcoming cellular generations.

## 1. Introduction

Wireless and mobile applications have experienced significant growth, evolving from analog communication to digital communication. Modern antenna designs face challenges related to space constraints, interoperability, supporting multiple frequency bands, adhering to specific absorption rate (SAR) regulations, and accommodating hearing aid compatibility. Additionally, digital communication requires addressing issues such as variable data rates, high capacity, and scalable bandwidth at base stations and mobile devices. MIMO antenna behavior is characterized by several parameters. Far-field gain measures the intensity of radiation in the far-field region. Diversity gain quantifies the improvement in signal quality achieved through multiple antennas. The envelope correlation coefficient assesses the correlation between different antenna elements. The total active reflection coefficient measures reflections and losses in the antenna system. Mean effective gain evaluates the average gain in a particular direction. 

Next-generation mobile networks (5G, B5G) provide much faster connection and considerably higher data rates compared to fourth generation (4G) systems with better stability, higher channel capacity, more spectral efficiency, and lower energy consumption. International Telecommunication Union identifies new radio (NR) for International Mobile Telecommunication (IMT) NR FR-I (sub-6-GHz Band) with channels n78 (3.3–3.8 GHz), n-77 (3.3–3.8 GHz), n79 (4.4–5 GHz), FR-II (24.25 GHz to 52.6 GHz) with channels in FR-2 being n-258 (24.25–27.5 GHz), n-257 (26.5–29.5 GHz), n260 (37–40 GHz), and n261 (28.35 GHz) [1]. The overall number of mobile subscribers reached roughly 8.4 billion in Q4 2022, with a net gain of 39 million. Nigeria had the largest net additions (+4 million) during the quarter, followed by the Philippines (+4 million) and Indonesia (+3 million). The global mobile subscriber penetration rate was 106%. Mobile broadband subscriptions increased by roughly 80 million in the third quarter, totaling 7.2 billion, a 5% rise year on year. Mobile broadband makes up about 86% of all mobile subscribers. 5G subscriptions increased by 136 million during the third quarter, bringing the total to slightly over 1 billion [2,3]. A total of 235 communication service companies have commercialized 5G services, and around 35 have constructed or launched 5G standalone (SA) networks. Refs. [2,3,4]. From Q3 (quarter 3rd) 2022 and Q4 (quarter 4th) 2022, mobile network data traffic increased by 10% quarter on quarter. Monthly worldwide mobile network traffic volume totaled 118 EB. In absolute terms, this implies that mobile network data traffic has more than quadrupled in only two years [5]. 

**Figure 1:**
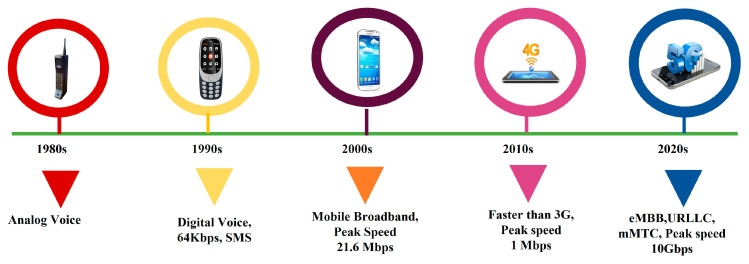
Evolution of cellular communication generation.

The massive volume of data traffic worldwide has increased manifold, so conventional antennas such as PIFA and monopoles cannot meet the demand. New multiple antenna technology with array and MIMO antenna is a promising technology to provide better channel capacity with additional bandwidth or transmission power. MIMO and m-MIMO are the next generation technology for mobile communication which groups multiple elements at the transmitter (Tx) and receiver (Rx) which provide better spectral efficiency, higher data rate, and channel capacity [6,7]. Figure 1 shows the evolution of cellular technology from 1G to 5G. With the introduction of first generation (1G) cellular networks in the 1980s, cellular technology has experienced substantial progress. With each new generation, network speed, capacity, and functionality have improved, enabling more advanced mobile devices and apps.

Evolution of cellular technology 1G–5G:

First generation (1G) networks were analog and only supported voice calls. These networks were subject to interference and espionage because they employed frequency modulation (FM) to transmit signals. In 1979, Japan launched the first 1G network. Second generation (2G) networks were the first digital cellular networks that were developed in the early 1990s. To boost the capacity and quality of voice calling, these networks employed TDMA and CDMA technologies. Short messaging service (SMS) allows subscribers to send or receive text messages and was also deployed on 2G networks. Third generation (3G) networks were developed in the early 2000s and significantly improved network speed and data capacity. These networks enabled mobile internet access, video calling, and multimedia messaging by utilizing HSPA (High-Speed Packet Access) and WCDMA (Wideband Code-Division Multiple Access) technologies. Fourth generation (4G) networks, introduced in the late 2000s, provided faster network speeds and more data capacity than 3G networks. Long-term evolution (LTE) technology was utilized in these networks to enable faster download and upload rates, lower latency, and improved coverage. More sophisticated UE applications, such as online gaming, video streaming, and cloud computing, were also made possible by 4G networks. Fifth generation (5G) networks, which debuted in the 2010s, are the most recent iteration of cellular technology. The 5G networks employ modern radio technology including millimeter wave (mmWave) and massive MIMO to enable faster network speeds, reduced latency, and more data capacity than 4G networks. They also allow new applications such as virtual and augmented reality, smart cities, and self-driving cars. Major 3GPP (3rd generation partnership project) releases for the evolution of 5G from advanced LTE are given as follows [8]:

- 3GPP Release 15: This was the first release of the 5G standard, published in December 2017. It introduced the 5G New Radio (NR) technology and defined the initial specifications for 5G networks.
- 3GPP Release 16: This is the second major release of the 5G standard, published in September 2020. It introduced enhancements, new features for 5G NR and the 5G core network, as well as specifications for new use cases and applications.
- 3GPP Release 17: This is the next major release of the 5G standard, currently under development and expected to be published in 2022. It will continue to build on the capabilities and features introduced in previous releases, while also introducing new enhancements and use cases.
- 3GPP Release 18: This is the next major release of the 5G standard, currently under development and expected to be published in 2023. It will further expand the capabilities and potential use cases for 5G technology, enabling new services and applications and enhancing the performance, security, and reliability of 5G networks. The cellular generation evolution along with the characteristics is summarized in Table 1 [9].

In this survey, detailed analysis of the most widely used MIMO antenna configurations, wideband/ultrawideband MIMO antennas, multiband/reconfigurable MIMO antennas, and circularly polarized MIMO antennas is presented. A detailed comparison of the characteristics of MIMO antennas is also presented for various wideband, multiband, and circularly polarized MIMO antennas. The study highlights the different design approaches for efficient MIMO antennas for 5G systems. The compactness of handheld devices and the gadgets used in modern wireless communication pose significant challenges in terms of antenna size and mutual coupling reduction when placing multiple antennas in such scenarios. When multiple antennas are placed in close proximity, mutual coupling between them becomes a critical issue. The study highlights the different isolation or decoupling techniques used in the literature and their effectiveness in reducing mutual coupling among different ports in MIMO antennas with limitations and advantages. Firstly, the survey highlights the several techniques to design wideband/ultrawideband antennas and presents a comparative analysis based on their performance parameters such as gain efficiency, isolation, and diversity parameters of MIMO antennas. Secondly, multiband/reconfigurable antenna approaches are discussed where reconfigurability is achieved using switching action based on PIN diode and multiband using characteristic mode analysis, slots, defective ground structure, partial ground plane, or EBG (electronic bandgap structure) or other periodic structures such as FSS (frequency selective surfaces). Finally, the design approaches of the circular polarized MIMO antennas for LHCP/RHCP or bidirectional or with the polarization diversity are discussed in detail.

The introduction part in Section 1 gives a glimpse into the cellular communication evolution, the essence of the MIMO antenna in 5G or future generation wireless communication, and important 3GPP releases related to 5G. Section 2 presents a key enabling technology of 5G as compared to conventional earlier wireless generation. Section 3 presents a brief introduction to diversity parameters envelope correlation coefficient (ECC), channel capacity, mean effective gain (MEG), and spectral efficiency for MIMO antenna. In Section 4, advantages of the MIMO antennas as compared to conventional SISO, MISO, or SIMO antenna systems are discussed. Section 5 focuses on different MIMO antenna design approaches along with isolation techniques used and comparison based on different antenna characteristics. Section 6 focuses on the outcome of the review based on recent trends, limitations, advantages, and future aspects of the design of MIMO antennas for WB/UWB, reconfigurable/multiband, and circularly polarized (CP) with decoupling techniques. Section 7 focuses on the design challenges for the MIMO antennas and finally Section 8 specifies the conclusion of the survey.

## 2. Key Features of 5G

In the context of 5G, the term “key enablers” refers to the fundamental technologies or components that are essential for the successful establishment and functioning of 5G networks [10,11,12]. These enablers play a vital role in providing the required capabilities and features that make 5G feasible, allowing it to deliver its distinct characteristics and advantages.

### 2.1. Millimeter-Wave (mmWave)

**Figure 2:**
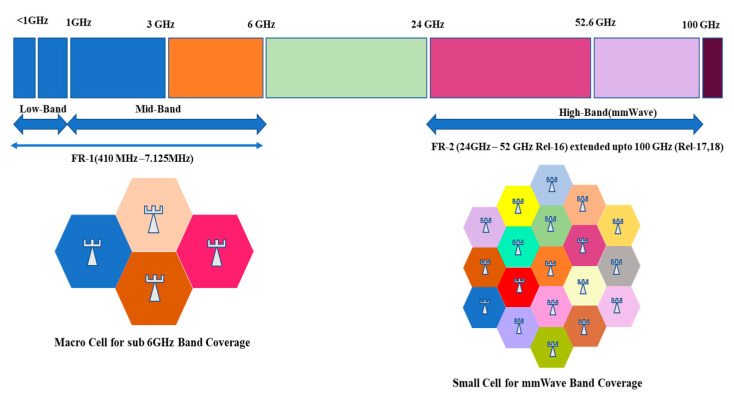
5G NR FR1 and FR2 frequency spectrum with cell size requirement.

Millimeter-wave (mmWave) [13] technology is a key technology for fifth generation (5G) cellular communication. It uses radio frequencies in the NR FR-I (410 MHz to 7.125 MHz) and FR-II (24 GHz to 52 GHz) range, which covers mmWave bands as shown in Figure 2. These higher bands allow for increased data transfer rates, lower latency, and increased capacity. The challenge of using mmWave technology is that these higher frequencies have shorter wavelengths, which means that the signals are more prone to fading by objects and hindered by obstacles such as buildings and vegetation. To overcome this challenge, 5G networks that use mmWave technology typically require more base stations or access points, which are installed in closer proximity to one another.

Another issue is that mmWave signals are more susceptible to interference from other wireless signals as well as meteorological conditions such as rain and fog. To mitigate these issues, 5G networks that use mmWave technology use advanced signal processing techniques and beamforming to focus the signals in specific directions, which improves the quality of the signal and reduces interference. Despite these challenges, mmWave technology offers significant benefits for 5G networks, including faster data transfer rates, lower latency, and increased capacity. These benefits are particularly important for applications that require high bandwidth, such as VR (virtual reality), AR (augmented reality), and HD video streaming.

### 2.2. MIMO/m-MIMO

**Figure 3:**
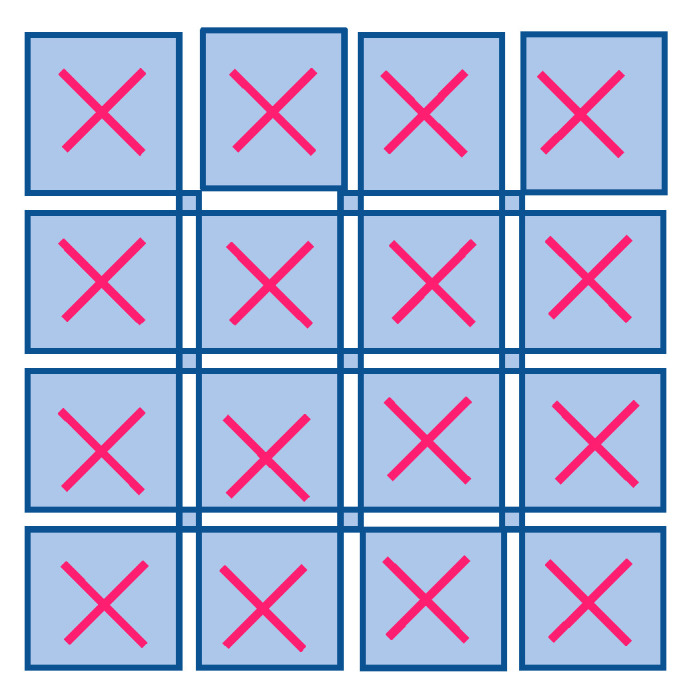
16T × 16R MIMO antenna array (where X denotes transceiver).

m-MIMO technologies are another key technology for 5G cellular communication. It makes use of multiple antennas for the transmission and reception of signals simultaneously, which increases the network’s capacity and coverage. SISO, SIMO, or MISO has also been employed for the transmission and reception of signals in a traditional wireless communication system. However, with Massive MIMO technology, multiple antenna-elements are used on both sides of the transmitter and receiver system. The 16T × 16R MIMO antenna array is shown in Figure 3 below. This allows for multiple signals to be transmitted and received simultaneously, which increases the network’s capacity and coverage [14].

Massive MIMO technology works by using complex signal processing algorithms to separate the different signals that are being transmitted and received by the multiple antennas. This allows telecommunication companies or regulatory bodies to efficiently use the existing spectrum and reduces interference between different signals. Massive MIMO technology not only boosts capacity and coverage but also enhances the quality of the wireless signal.

### 2.3. Small Cells

With the advancement of networks and the growing need for data traffic, deploying small cells has emerged as a viable solution. These small, low-power wireless access points, operating within the licensed spectrum and managed by operators, offer improved cellular coverage and capacity. They are particularly beneficial in addressing coverage gaps, maintaining service quality, and efficiently utilizing spectrum resources. Small cells address this issue by providing additional coverage and capacity in areas where the large cell towers are not effective. Small cells can be of micro, macro, femto, or metro type. In urban areas, micro and metro type small cells offer coverage up to a few hundred meters. Pico cells provide coverage within a few tens of meters, both indoors and outdoors, primarily in public areas such as shopping malls, airports, and railway stations. Femtocells are mainly utilized in residential areas, covering a range of a few tens of meters.

These small cells are installed on streetlights, utility poles, and other existing infrastructure in urban environments using a large number of access points (APs). By using small cells, 5G wireless networks can provide improved coverage and capacity in dense urban environments, which is essential for many applications such as smart cities, autonomous vehicles, and IoT devices. There are some challenges with femtocells and microcells such as management of the resources through scheduling at the edge of cell boundary, static and dynamic spectrum allocation, and frequent handover in some scenarios [15,16].

### 2.4. Network Slicing

**Figure 4:**
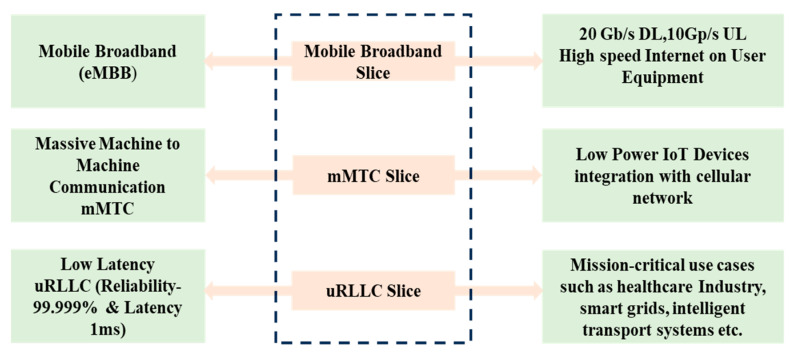
Network slicing feature of 5G for different use cases.

Network slicing is another feature of a 5G wireless network that allows many virtual networks to be formed within a single physical network, enabling telecom operators to deliver customized services to users as per the scenario shown in Figure 4. In a traditional wireless network, all the users share the same network resources, which can limit the capability of the network to sustain a wide range of services with varying requirements for bandwidth, latency, and reliability. A network slice is a distinct and isolated network portion designed to be self-contained and secure. It is created by logically separating resources to fulfil specific user needs, taking into account the quality of service (QoS) provided by the slice. The concept of network slicing enables efficient allocation of network resources, resulting in cost optimization and the ability to meet diverse requirements. In essence, network slicing allows for the customization and fine-tuning of network capabilities to best suit the individual needs of different users or applications. Network slicing enables operators to create virtual networks with unique resources and characteristics to support diverse services and applications. Each network slice is tailored to meet the specific needs of a particular facility or application, such as low latency for autonomous vehicles or high bandwidth for video streaming. Network slicing is a key feature for many 5G use cases, such as smart cities, industrial automation, and healthcare. It enables operators to provide customized facilities to different users and applications, and it enables the network to support a broad range of services with varying requirements for bandwidth, latency, and reliability [17,18].

### 2.5. Cloud Computing

5G and B5G make use of cloud computing, which has another advantage for wireless communication. It refers to delivering computing services over the cloud, including servers, storage, applications, and other resources, which can be accessed remotely by users and devices. In a 5G network, cloud computing provides numerous services and applications to users and devices, including edge computing, AI, and IoT applications. Cloud computing enables these services and applications to be delivered in a scalable, efficient, and cost-effective manner, which is essential for supporting the massive data volumes and high-performance requirements of 5G wireless networks. Cloud computing also enables AI and ML algorithms to be run on large datasets in a scalable and cost-effective manner. Machine learning provides numerous benefits, such as its capacity to analyze enormous datasets and detect patterns and trends [19,20]. This capability allows for precise predictions of future occurrences. Another advantage is that machine learning algorithms can independently make decisions and evolve over time, continually improving their performance without human involvement. Moreover, these algorithms excel at processing intricate and multidimensional data, even in uncertain and dynamic environments akin to those encountered in 5G/6G scenarios. Additionally, machine learning algorithms demonstrate accelerated learning speeds, especially when confronted with large-scale problems. This is essential for many 5G applications, such as autonomous vehicles and industrial automation, which require real-time decision-making and analysis of large amounts of data.

### 2.6. Virtualization

This refers to the creation of virtual resources, such as servers, storage, and networks, that can be accessed and managed independently of the physical resources on which they are based. In a 5G network, virtualization is used to create virtualized network functions (VNFs) and virtualized network elements (VNEs), which enable operators to provide different types of services and applications in a scalable and flexible manner. Virtualization allows network functions to be run on standard hardware, rather than specialized hardware, which can reduce costs and improve scalability. Virtualization in a 5G network can support network slicing which is a key advantage. Network slicing involves creating multiple virtual networks within a single physical network, each with its own set of resources and characteristics, to support different types of services and applications. Operators can allocate resources to users on dynamic bases with a virtualization function for different services and applications in real-time, based on their changing requirements. This enables the network to be more efficient and responsive to changing traffic patterns, which can improve performance and reduce costs.

### 2.7. Edge Computing

Edge computing-oriented communications have emerged as promising technologies for enabling 5G/6G advancements. The rapid growth of smart devices and data traffic has driven operators to explore sustainable alternatives, such as cloud computing and virtualization, for deploying numerous base stations. Cloud-RAN (C-RAN) is an innovative RAN technology that leverages virtualization concepts, offering on-demand access to scalable computing resources from a shared pool [19,20]. It refers to the processing of data at the edge device, closer to the user, rather than in a centralized data center. This reduces the amount of time required to process and transmission of data over the network, which can improve performance and reduce costs. Edge computing involves placing resources, such as servers and storage devices, closer to the user, in proximity to the radio access network (RAN) or base station. This enables data to be processed and analyzed in real-time, at the edge of the network, rather than being transmitted to a centralized data center for processing. By operating as an intermediary, the edge cloud optimizes backhaul bandwidth by forwarding data to the core network only when necessary. This is essential for many 5G applications, such as autonomous vehicles and industrial automation, which require immediate response to changing conditions.

### 2.8. Carrier Aggregation (CA) and Beamforming

**Figure 5:**
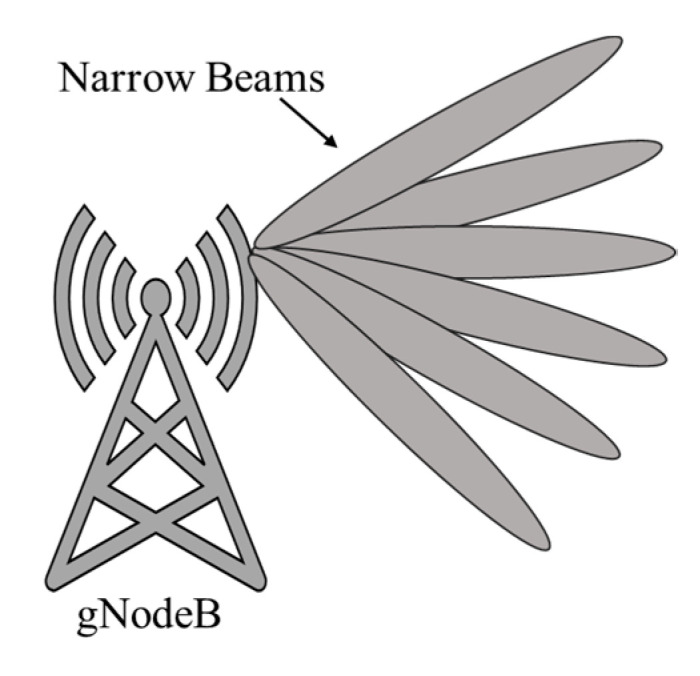
Narrow beam-forming at gNodeB in 5G cellular communication in massive MIMO.

Carrier aggregation involves the combination of multiple frequency bands to create a wider frequency band, which can provide higher data rates and better performance [21,22]. This enables operators to make more efficient use of the available spectrum and provide a higher quality of service to users. Beamforming involves the directional transmission and reception of signals between a base station and user equipment. It uses multiple antennas to create narrow beams that can be directed toward specific users or devices, rather than broadcasting signals in all directions as shown in Figure 5. This enables operators to provide higher data rates, better coverage, and more efficient use of the available spectrum. Beam-forming is a critical technology for millimeter wave (mmWave) frequencies, which are used in some 5G networks to provide extremely high data rates. mmWave frequencies have high propagation losses, which means that they are easily absorbed by objects in their path, such as buildings and trees. Beamforming enables operators to overcome these propagation losses by directing the signal towards specific users or devices, rather than broadcasting it in all directions.

## 3. MIMO Diversity Parameters

The MIMO diversity parameters, including the envelope correlation coefficient (ECC), total active reflection coefficient (TARC), channel capacity, mean effective gain (MEG), and spectral efficiency play crucial roles in the performance and effectiveness of MIMO antennas. These parameters collectively contribute to the performance, reliability, and efficiency of MIMO antennas. By optimizing these parameters, MIMO systems can achieve higher data rates, improved signal quality, enhanced system capacity, and better utilization of available wireless resources, ultimately enabling advanced wireless communication applications.

### 3.1. Envelope Correlation Coefficient (ECC)

In MIMO systems, evaluating the coupling between radiating elements is crucial, and ECC serves as a vital parameter for this purpose. Unlike isolation parameters, ECC considers return-loss of all ports and isolation among different ports to characterize complete antenna mutual coupling. The recommended value of ECC by the ITU should be less than or equal to 0.5 for mobile communication systems. Better performance in MIMO systems is indicated by a low ECC value, which signifies reduced coupling between radiating elements. Conversely, a higher ECC value can have an adverse effect. ECC for higher radiation efficiency greater than 90% can be obtained directly from the s-parameter and given as [23,24]:

(1) $$\left|\rho_{ECC}\left(k,l,P\right)\right|=\frac{\sum_{m=1}^{P}S^{*}{}_{k.m}S_{m,l}}{\sqrt{\prod_{s\left(=k,l\right)}\left\{1-\sum_{m=1}^{P}S^{*}{}_{k,m}S_{m,s}\right\}}}$$

where k = 1 to m, l = 1 to m, m = mth port, s = sth port, and P = number of antennas.

To calculate ECC for an antenna with radiation efficiency below 90% involves the far-field parameters of the radiating antenna [24].

(2) $$\rho_{ECC}=\frac{\iint {\left[\left(\bar{F_{1}}(\theta ,\emptyset \right)\cdot \left(\bar{F_{2}}(\theta ,\emptyset \right)\right]}^{2}d\Omega }{\iint {\left(\bar{F_{1}}(\theta ,\emptyset \right)}^{2}d\Omega \iint {\left(\bar{F_{2}}(\theta ,\emptyset \right)}^{2}d\Omega }$$

### 3.2. Total Active Reflection Coefficient (TARC)

TARC is a parameter of the MIMO antenna to validate the scattering parameters (s-parameters) for diversity performance. TARC employs both random signals and phase angles for diagonal and adjacent antenna ports to determine the behavior of s-parameters such as s11, s12, and s13 for particular phase combinations between the ports. TARC is calculated using the incident vector ai and reflected vector bi, which is independent and identically distributed Gaussian random variables. TARC can be calculated by [23,24]:

(3) $$\Gamma_{x}^{t}=\sqrt{\frac{\sum_{i=1}^{P}{\left|y_{i}\right|}^{2}}{\sum_{i=1}^{P}{\left|x_{i}\right|}^{2}}}\;$$

where $x_{i}$ is the incident signal at the *i*th port and $y_{i}$ is the reflected signal at the *i*th port and P is the total number of ports. For p-port, the scattering matrix is given by [23]:

(4) $$\left[\begin{array}{c} y_{1}\\ y_{2}\\ y_{p} \end{array}\right]=\left[\begin{array}{ccc} s_{11} & s_{12} & s_{1n}\\ s_{21} & s_{22} & s_{2n}\\ s_{n1} & s_{n2} & s_{nn} \end{array}\right]\left[\begin{array}{c} x_{1}\\ x_{2}\\ x_{p} \end{array}\right]\;$$

By solving the given scattering matrix for the scattering/reflected parameter, TARC can be obtained as [23]:

(5) $$\Gamma_{x}^{t}=\frac{\sqrt{{\left|\left(s_{ii}+s_{ij}*e^{j\theta }\right)\right|}^{2}+{\left|\left(s_{ji}+s_{jj}*e^{j\theta }\right)\right|}^{2}}}{\sqrt{P}}$$

where θ is the phase angle between diagonal and adjacent ports in MIMO configuration. The values of θ vary for the different ports to see the actual effect on ports.

### 3.3. Channel Capacity

**Figure 6:**
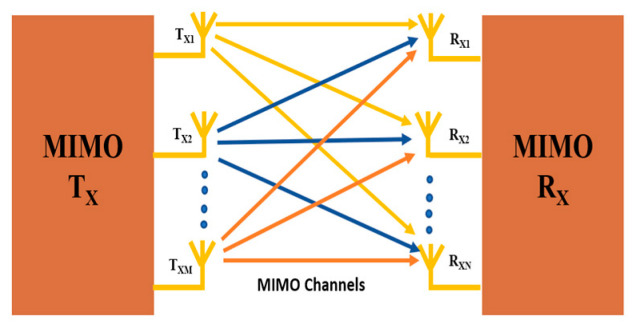
MIMO transmitter, receiver, and channel.

Compared to SISO antenna systems, MIMO antennas exhibit higher channel capacity, which is a crucial performance parameter as given by [25,26,27]. The MIMO T_x_/R_x_ block diagram with MIMO channel is shown in Figure 6.

As per Shannon capacity theorem, the capacity for the SISO system is given as [25]:

(6) $$C=B_{t}\;log_{2}\left(1+SNR\right)$$

where *B_t_* is the available total bandwidth and *C* is the Shannon channel capacity. The channel matrix “*H*” in the MIMO system is expressed based on the number of transmitter and receiver antennas, denoted as “*m_t_*” and “*m_r_*”, respectively [26].

(7) $$H=m_{t}*m_{r}$$

Let $E_{S}$ is the average symbol energy, the received signal at the receiver is given as [26]:

(8) $$y\left(t\right)=\sqrt{\frac{E_{s}}{m_{t}}}\;Hs\left(t\right)+n\left(t\right)$$

where *y*(*t*) is the received signal vector of $m_{r}*1$, *s*(*t*) is transmitter signal vector of $m_{t}*1$, *n*(*t*) spatial-temporal white noise with zero mean and variance $N_{0}$. The average power in terms of the covariance matrix of *s*(*k*) is given as: $R_{ss}=ss^{H}$(without time index).

Case 1: Let the wireless channel be deterministic and known to the receiver, then the capacity of the MIMO system is given as [26]:

(9) $$C=B\;log_{2}\;Det\left(I_{m_{r}}+\frac{E_{s}}{m_{t}N_{0}}\;HR_{ss}H^{H}\right)bits/sec$$

where $E_{s}$ is symbol energy, $N_{0}$ noise spectral density of Gaussian white noise, $I_{m_{r}}$ is the identity matrix of $m_{r}*m_{r}$ order, and $\frac{E_{s}}{m_{t}N_{0}}$ is the signal-to-noise ratio (SNR).

Case 2: when the transmitter does not know the channel, then the s vector may be chosen as $R_{ss}=I_{m_{t}}$, where $I_{m_{t}}\;$is the identity matrix, then the capacity of the MIMO system is [26]:

(10) $$C=B\overset{r}{\underset{i=1}{\sum }}log_{2}\left(1+\frac{E_{s}}{m_{t}N_{0}}\lambda_{i}\right)bits/sec$$

where the channel matrix has rank r and the capacity is the addition of ‘r’ SISO channels with the power gain $\lambda_{i}$ where i=1, 2,……r. Let $m_{t}=m_{r}=m$, then rank r=m, in this case, maximum capacity is achieved when the channel matrix H is an orthogonal matrix i.e., $H^{H}H=HH^{H}$ [26]:

(11) $$C=B\;M\;log_{2}\left(1+\frac{E_{s}}{N_{0}}\right)\;bits/sec$$

### 3.4. Mean Effective Gain (MEG)

MEG refers to the combined gain of all the elements in the MIMO system, and it is typically expressed in decibels (dB). Mean effective gain is a measure of the effective gain of an antenna in a multiple-input multiple-output wireless communication network. MEG takes into account the antenna efficiency, the power patterns of the antennas, and the spatial correlation of multiple antennas used in the MIMO system [28].

(12) $$MEG=\iint_{\emptyset =0,\theta =0}^{\emptyset =2\pi ,\theta =\pi }\left[\frac{XPR}{1+XPR}G_{\theta }\left(\theta ,\phi \right)P_{\theta }\left(\theta ,\phi \right)+\frac{1}{1+XPR}G_{\phi }\left(\theta ,\phi \right)P_{\phi }\left(\theta ,\phi \right)\right]d\Omega$$

where dΩ(solid angle)=sin θ dθdϕ, XPR is the cross-polarization power ratio, $G_{\theta }\left(\theta ,\phi \right),G_{\phi }\left(\theta ,\phi \right)$ are power gain of antennas, and $P_{\theta }\left(\theta ,\phi \right)$, $P_{\phi }\left(\theta ,\phi \right)\;$ are the angular density functions of arriving radio waves.

The above equation requires a 3D radiation pattern of user equipment but the calculation of MEG using a 3D radiation pattern is tricky; therefore, using a 2D radiation pattern for the calculation of MEG is participable and is given in the following equation [28]:

(13) $$MEG=\overset{2\pi }{\underset{0}{\int }}\left[\frac{XPR}{1+XPR}G_{\theta }\left(\frac{\pi }{2},\phi \right)P_{\theta }\left(\frac{\pi }{2},\phi \right)+\frac{1}{1+XPR}G_{\phi }\left(\frac{\pi }{2},\phi \right)P_{\phi }\left(\frac{\pi }{2},\phi \right)\right]d\phi$$

Other equations for MEG using the s-parameter can be [28,29]:

(14) $$MEG_{ith\;port}=0.5\left\{1-\overset{N}{\underset{j=1}{\sum }}{\left|s_{ij}\right|}^{2}\right\},where\;N=number\;of\;antenna,\;j=j\mathrm{th}\;port$$

### 3.5. Spectral Efficiency

It is a measure of the amount of data that can be transmitted over a radio spectrum. It is generally expressed in bits per second per hertz (bps/Hz). Spectral efficiency (bits/s/Hz) is defined as $\eta =\frac{R}{B_{t}}$, where η - spectral efficiency, R is bit rate, and Bt is available total bandwidth. When using SISO (single input single output) technology, the spectral efficiency of 4G-LTE is 4.08 bits/s/Hz, which means that 4.08 bits of data can be transmitted per second per hertz of the spectrum. However, when using 4 × 4 MIMO technology, the spectral efficiency increases to 16.32 bits/s/Hz, which is four times higher than SISO [23].

## 4. Advantages of MIMO Systems

The growing range of mobile applications has led to a demand for Gigabit data rates to cater to users with different mobility levels, including both low and high-mobility scenarios. To address this requirement, traditional single antennas in mobile devices are being replaced with MIMO antennas. By implementing MIMO technology, mobile devices can deliver enhanced quality of service, offering uninterrupted signaling, high data rates, increased capacity, and improved spectral efficiency. Some of the advantages are listed below.

### 4.1. Increased Data Rates

MIMO technology allows for higher data rates compared to traditional single-input single-output systems, as multiple data streams can be transmitted simultaneously over the same frequency band. As the number of MIMO layers increases, the overall throughput of the system also increases.

### 4.2. Improved Signal Quality

**Figure 7:**
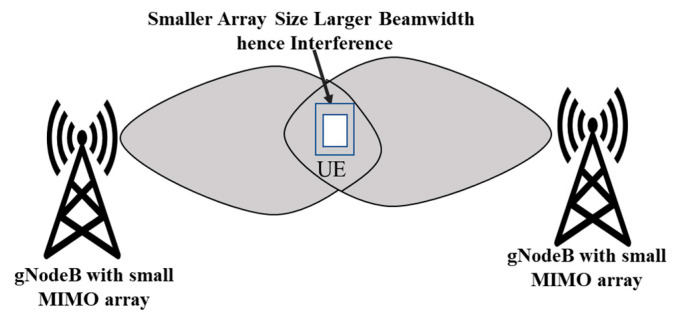
The smaller the array size and large beamwidth, the more interference at the coverage boundary.

**Figure 8:**
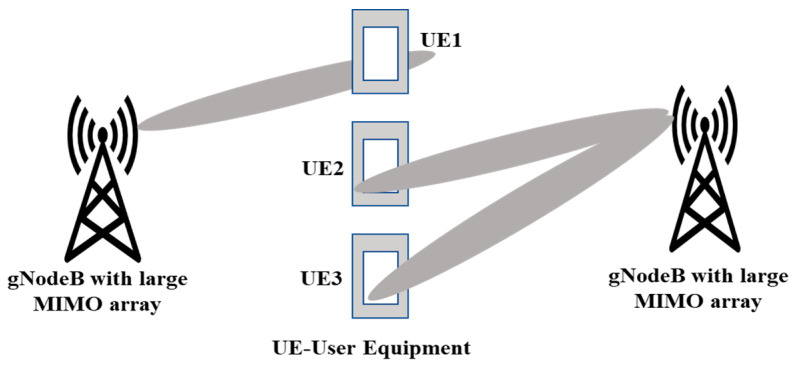
Larger array size sharper beam low interference.

MIMO technology helps to improve signal quality by reducing the effects of fading, interference, and noise. As conventional SISO (single input single output), a single stream of data will be transmitted between transmitter and receiver which results in more interference and fading effects at the coverage boundary due to the large beamwidth of base station antennas shown in Figure 7, whereas MIMO or massive MIMO at gNodeB (base station antenna in 5G) generates a more directional beam, which improves the coverage and reduces the effects of interference at the coverage boundary as shown in Figure 8.

### 4.3. Increased Range and Coverage

**Figure 9:**
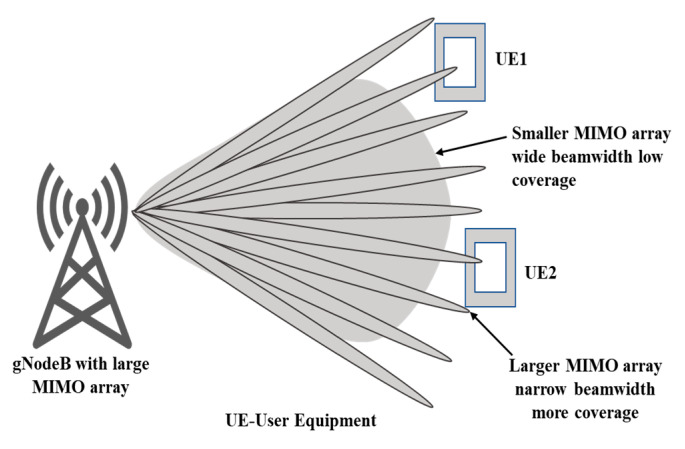
Directional beams have a stronger coverage area and improved signal-to-noise ratio.

MIMO technology can extend the range and coverage of wireless networks by improving link quality and reducing the likelihood of signal dropouts. Figure 9 shows that a larger MIMO array or massive MIMO generates a more directional beam which can improve the coverage area as compared to a conventional SISO system.

### 4.4. Better Spectral Efficiency

MIMO technology enables better spectral efficiency which allows more effective use of the available radio spectrum. This is achieved by transmitting or receiving multiple data streams simultaneously.

### 4.5. Compatibility with Existing Standards

MIMO technology is retrograde compatible with existing wireless standards, which means that it can be easily integrated into existing wireless networks without any major changes in the infrastructure.

## 5. MIMO Antenna Design Approaches for FR-1 and FR-2 Bands

**Figure 10:**
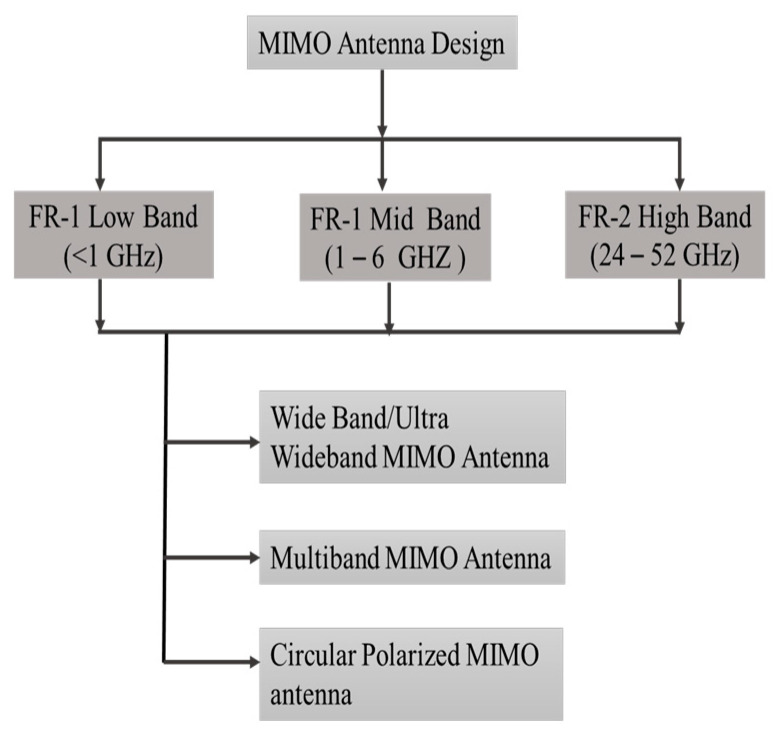
MIMO antenna design based on FR-1 and FR-2 band as per ITU.

This section discusses various MIMO antenna designs such as wideband, multi-band, and circular polarized antenna configurations for FR-1 and FR-2 bands. These designs aim to achieve wide frequency coverage, support multiple frequency bands, and enable efficient circular polarization to meet the diverse requirements of wireless communication systems in these frequency ranges as shown in Figure 10.

### 5.1. Wideband/Ultra-Wideband MIMO Antenna Design Approaches with Decoupling Structure

**Figure 11:**
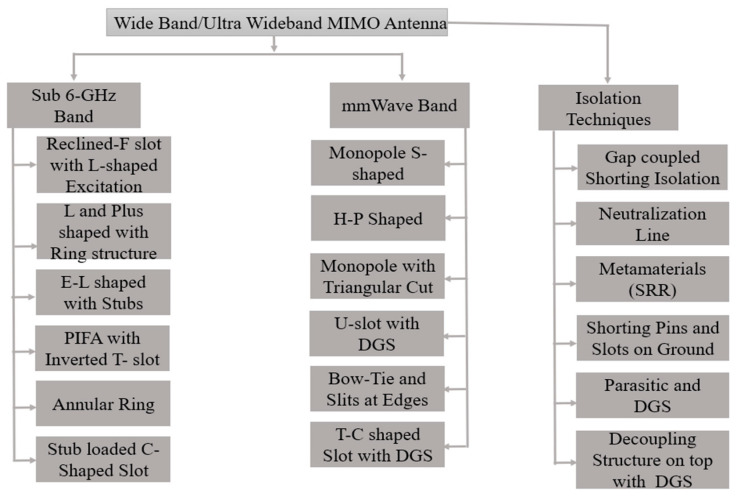
WB/UWB-MIMO antenna design approaches with different isolation techniques.

Wideband MIMO antennas are designed to operate across a broad frequency range, typically spanning multiple frequency bands within FR-1 and FR-2. These antennas employ various techniques to achieve wide frequency coverage, such as broadband feeding networks and radiating elements. Wideband MIMO antennas are capable of supporting multiple frequency bands simultaneously, providing enhanced spectral efficiency and compatibility with different wireless communication standards. The categorization of wideband MIMO antenna for sub-6GHz band and mmWave are shown in Figure 11.

In article [29], MIMO antenna employs defected ground structure (DGS) and defected microstrip structure (DMS) approaches to decrease mutual coupling and cross-polarization in the mm-wave band, n258 ranges from 24.25 to 27.5 GHz, with stub-loaded CPW structures with elliptical radiators. The use of a metallic plane with defects as a reflector on the substrate’s backside improves co-polarization to cross-polarization isolation and radiation characteristics, also achieving isolation of 35 dB and co-polarization to cross-polarization isolation of greater than 20 dB. MIMO performance parameters such as envelope correlation coefficient (0.1 × 10^−6^), diversity gain of 10 dB, and CCL of 0.005 bps/Hz were obtained. This study [30] describes a four-port S-shape MIMO wideband millimeter wave antenna ranging from 25 GHz to 39 GHz which covers NR FR-II band n258, n257, n261, and n260. The orthogonal orientation of radiating elements along with the line resonator reduces mutual coupling which yields 26 dB of isolation among ports. A tilted square patch with extended arms is added to the top of a substrate line resonator to reduce coupling with the other ports. The maximum gain of 7.1 dBi, ECC lower than 0.05, and efficiency greater than 85% were reported. A reclined-F slot and L-shaped excitation were etched on a shorted rectangular patch which results in multi-mode resonance and broad bandwidth of 88% was achieved. Single antenna design was converted into 8 × 8 MIMO, which is fabricated on the bezels of the smartphone. The antenna in [31] has efficiencies ranging from 40% to 75% which covers the full 5G NR spectrum (n77/n78/n79) and LTE band. The antenna has isolations among ports above −10 dB, and ECC less than 0.07. This investigation describes an L-shaped slot with a plus-shaped structure on a patch enclosed in a circle [32], while the bottom layer is partially covered by a ground plane. The design was further extended to 8-port MIMO for 5G MIMO applications with effective impedance bandwidth of 3.05 to 3.74 GHz. MIMO performance parameters such as isolation over −15 dB and ECC less than 0.1, respectively, achieved. A four-port wide band EL slotted radiated patch [33] with two stubs extended to MIMO configuration was designed for 5G NR in sub-6 GHz and 5 GHz WLAN bands. This configuration makes use of the partial ground to achieve wideband operation with effective bandwidth ranges from 3.20 to 5.85 GHz. Incorporating a novel UPMS decoupling structure between radiating patches resulted in a significant improvement in isolation gain of 17.5 dB and high efficiency of 85% across desired frequency bands. Another HP-shaped 4 × 4 wideband MIMO antenna [34] for mmWave frequency ranges from 36.83 to 40 GHz with an effective gain of 6.5 dB reported. Anti-parallel position and diagonal arrangement of the antenna without decoupling network isolation gain of −45 dB was achieved and spectral efficiency of 0.6 bits/s/Hz with excellent EEC value below 0.001. The study presented a PIFA antenna with an inverted T-shaped slot that utilizes multimode theory to achieve wideband coverage across 5G New Radio bands n77/n78/n79 and LTE bands. For decoupling network, a shorting stub is used. Another similar 8 × 8 MIMO configuration for mobile phones with an antenna on the mobile phone bezel’s front and back side covered sub-6-GHz band ranges from 3.3 to 7.2 GHz was discussed [35,36]. Annular ring (AR) based [37] 4-port MIMO antenna for access point applications was discussed with effective bandwidth of 3.3–5.0 GHz, an efficiency of 84%, and an ECC less than 0.05 achieved. A GCS strip used as a decoupling network between ports and placed at 90 degrees apart at angle ϕ = 45°, 135°, 225°, 315° provide the short circuit between the patch and ground plane so that adjacent ports are separated by one GCS strip. A C-shaped transparent flexible 4-port MIMO antenna [38] was presented with circular stubs. AgHT-4 and Melinex substrates were used for optical transparency. An L-shaped partial ground plane with a C-shaped radiator resulted in a wide bandwidth. The antenna offers a sub-6 GHz bandwidth with 2.21 to 6 GHz and an isolation of over 15 dB across all ports. The antenna also provides a peak gain of 0.53 dBi with a minimum efficiency of 41%, which is suitable for a flexible structure. The manuscript [39] presented a UWB antenna design with an impedance bandwidth (IBW) of 2 to 18 GHz. The modified patch features a double U-slot, tapered edges, and a triangular opening, along with a rectangular strip on the partial ground plane. An effective gain between 2 and 6.5 dB and an efficiency of more than 70% was achieved. Five split ring resonator (SRR)-based unit cells arranged linearly between radiating patches used as the decoupling structure allow an isolation gain of −20 dB. The paper [40] introduced a wideband MIMO antenna based on metamaterials, designed for the 5th generation sub-6 GHz band and covering a frequency range of 3.27–3.82 GHz. The antenna’s performance was improved using a 4 × 4 meta surface unit cell, resulting in a significant increase in impedance bandwidth from 2.3% to 13.2%, along with increased efficiency from 83% to 92% and a peak gain of 8.1 dBi. The bottom of the square patch tapered with the circular curve, sandwiched between the ground plane and metal surface. The decoupling structure consists of slots in the ground and shorting pins which provide stable gain over a desired frequency band with isolation of more than 32 dB achieved. Furthermore, the MIMO performance parameters exhibit diversity performance, with an ECC of less than 0.001 and a DG of 9.99 dB. A meta-material-based MIMO antenna [41] consists of CMSPR (combined metamaterial structure and parasitic ring) unit cells and triple lines. Metamaterial is used as a superstrate on the patch in the x-y plane whereas other configurations of metamaterial are used as a CMSPR wall in the y-z direction. Metamaterial structure acts as an isolating structure or spatial filter which increases the performance of and reduces the mutual coupling of electromagnetic energy among multiple antennas in the MIMO system. The triple lines approach was utilized to enhance the bandwidth which resulted in the total effective bandwidth of 28 to 32 GHz in the mmWave band. The significant increase in gain from 11.8 dB (without metamaterial) to 17.1 dB at resonant frequency 30 GHz was achieved with metamaterial structure. Moreover, the isolation of less than 36.7 dB at 30 GHz was obtained.

In this study [42], the authors presented an 8-elements MIMO antenna. DGS along with parasitic elements were utilized to achieve wideband impedance ranges from 3.3 to 6.1 GHz and strong decoupling of 15 dB achieved. The performance of the antenna was analyzed using characteristic mode theory, revealing efficiencies exceeding 50% and reaching up to 78%. The ECC is less than 0.11, and the channel capacity ranged from 35.6 to 41.3 bps/Hz, slightly below the theoretical limit. A flexible four-port MIMO antenna [43] was designed by the author, featuring four octagonal rings etched within a T-shaped conducting layer. The substrate’s edges were surrounded by inverted L and E-shaped structures, cut at a 45-degree angle for improved performance. Octagonal rings were responsible for achieving a wide bandwidth range from 2.39 to 5.86 GHz. The optimal distance between antenna elements required no decoupling structure. Self-isolation among antenna elements achieved more than 17.5 dB with maximum efficiency of 85%. The maximum gain of 4 dBi, ECC below 0.05, and DG more than 9.8 dB were achieved. The study [44] describes a miniaturized and wideband MIMO antenna that employs a modified radiator and ground. The antenna achieved an IBW of 6.4 GHz (26.5 to 32.9 GHz), with an average gain of 5 dBi and a peak gain of 5.42 dBi at 27.8 GHz. The radiation efficiency of the antenna was above 84% over the operating bandwidth. However, the antenna’s CCL was below 0.5 bits/s/Hz. The antenna achieves a directive gain of 9.99 dB, indicating its suitability for highly directional applications. The paper [45] describes a multi-input multi-output antenna array that covered the 5G New Radio Bands (n77/n78/n79) and wireless-LAN 5 GHz bands with effective IBW (−6 dB) covering 3.2 to 6 GHz. The antenna had a total efficiency ranging from 38% to 83% and an ECC of less than 0.31 across the entire band. The antenna array achieved a high isolation of 12.6 dB without a decoupling network. The antenna design [46] described a novel WB self-decoupled 8-element MIMO antenna for 5th generation mobile communication. The antenna works on the principle of common mode and differential mode theory to analyze the self-decoupling principle and generates two resonant modes which result in wideband operation at 6dB bandwidth ranges from 3.3–5.0 GHz. Self-decoupling is achieved by a loop antenna-type radiator with the coupled line on the substrate. As per the theory of CM and DM, the isolation between ports is the sum of the reflection coefficient of both modes. When the reflection coefficient of CM and DM are equal, better decoupling performance is achieved. Furthermore, the MIMO antenna has isolation greater than 11.5 dB between ports within the desired band, with an overall ECC lower than 0.065 in the frequency band of interest. The antenna’s efficiency ranges from 63.0% to 86% across the entire frequency range.

The study [47] introduced a compact MIMO microstrip antenna (MSA) designed for X/Ku band applications. The antenna system incorporates a parasitic component to decrease mutual coupling and a complementary split ring resonator (S-CSRR) to enhance its performance. The antenna system operates at 7.8 GHz and 14.2 GHz and has IBW of 560 MHz and 600 MHz, respectively, with mutual coupling below −26 dB. The isolation gains at 7.8 GHz and 14.2 GHz are below −26 dB and −22 dB, respectively, and ECC values are 0.07 and 0.04 for both bands, respectively. The antenna has diversity gains greater than 9.8 dB for both bands and an efficiency of 80% across desired bands. This paper [48] introduced a partial ground plane-based 2-element MIMO circular monopole MSA with an inverted L-shaped stub to achieve wide bandwidth ranging from 3.4 to 3.8 GHz for a sub-6-GHz band. The antenna achieved dual-band operation by utilizing a PIN diode and L-shaped stubs. The frequency range for the two bands is 3.35–3.94 GHz and 4.99–5.16 GHz, respectively, with efficiency levels of 65% and 75%. The antenna also achieved isolation levels of −14.5 dB and −12.5 dB for the two frequency bands, respectively. Other key performance indicators include a peak gain of 2.68 dBi at 5.1 GHz, an ECC below 0.09, and a MEG of ±3 dB. A 3-element and 4-element MIMO MSA [49] was investigated with orthogonal orientation for 5G mmWave, with an inverted L-shaped patch with a partial ground plane resulting in a wide bandwidth of 26–40 GHz. The 3-element MIMO had two rectangular slots on the ground plane, which led to isolation greater than 15 dB. The 4-element MIMO had a circular ring interconnecting the ground plane, and a plus-shaped slot on the ground plane achieved isolation greater than 24 dB and an ECC lower than 0.02. The antenna achieved a maximum peak gain of 10.2 dBi at 26 GHz with an efficiency of more than 70%. The WB (wideband) DGS, circular CPW (coplanar waveguide) feed, and elliptical slot on circular patch MIMO antennas [50] were optimized for achieving high performance in the 5G New Radio bands n257/n258/n261, which are critical for mm-wave frequency. The circular ring-connected ground structure used in the design ensured high isolation levels of more than 20 dB and minimized interference between the ports. The effective impedance wideband bandwidth of 24.8 to 44.45 GHz was achieved with an efficiency of more than 85% across the desired band. The ECC values for the antenna elements were less than 0.007, and the observed average gain was more than 5 dBi. The 6-element MIMO antenna [51] achieved a −6 dB IBW of 2.3 to 3.09 GHz and 2.99 to 4.17 GHz while −10 dB IBW of 2.38 to 2.72 GHz and 3.17 to 3.84 GHz for ISM bands. The single antenna achieved a gain of 4 dBi, with a radiation efficiency of 85%. The average gain at the 2.5 GHz frequency band was greater than 4 dBi, while for the 3.5 GHz frequency band, it ranged between 4 and 6 dBi. The ECC for the desired frequency bands with the center frequency of 2.5 GHz and 3.5 GHz being less than 0.17 and 0.125, respectively, while DG (diversity gain) was more than 9 dB for both operating bands. The antenna design [52] featured a modified quadrangular radiating patch with ground plane slots that enable a wide bandwidth of 2.1 to 16.9 GHz across S, C, X, and Ku microwave bands. Additionally, two L-shaped stubs in the ground were incorporated to achieve 16 dB isolation in the desired frequency band. In terms of MIMO performance, the design achieved an ECC below 0.001, and a DG greater than 9.997 dB across the band, indicating good radiation characteristics. The comparison of wideband MIMO antennas for their performance characteristics is given in Table 2. 

### 5.2. Reconfigurable/Multiband MIMO Antenna Design Approaches

**Figure 12:**
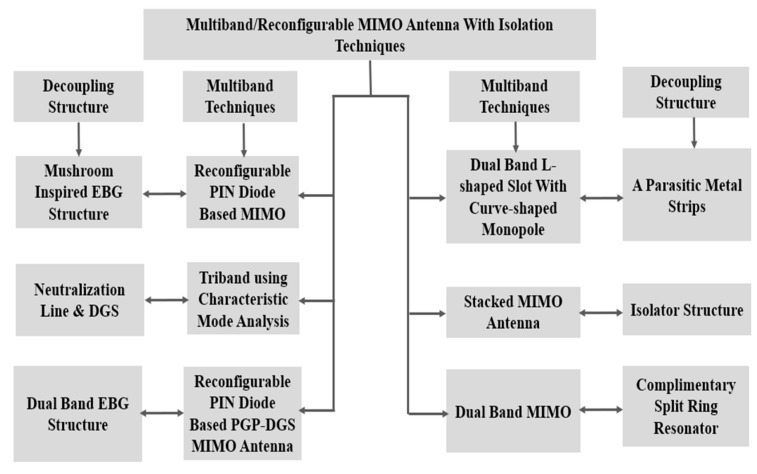
Reconfigurable and multiband MIMO antenna design approaches.

Reconfigurable/Multiband MIMO antennas are designed to support specific frequency bands. These antennas incorporate radiating elements or structures optimized for each desired frequency band. By utilizing frequency-selective elements, such as bandpass filters or resonant structures, multiband MIMO antennas can efficiently transmit and receive signals in each specific band. This design approach offers flexibility and compatibility with various network deployments and user equipment that may operate in different frequency bands as shown in Figure 12.

The study [53] presented a reconfigurable PIN diode-based equilateral triangular patch antenna for triple-band MIMO, suitable for L and S-band applications. An EBG structure integrated between two antenna elements on the substrate provided an isolation of more than 15 dB. The antenna resonated at frequency bands for different switching operations at 1.06/1.24 GHz, 1.52/2.13 GHz, and 2.19/2.63 GHz. Additionally, the antenna had an effective gain of over 3.8 dBi, and co-polarization and cross-polarization greater than 14 dB with ECC below 0.5 and diversity gain (DG) of 9.5 dB were achieved.

The study [54] described the analysis of tri-band (2.4–2.5 GHz, 3.3–3.6 GHz, and 4.8–5 GHz) MIMO antenna using characteristic mode theory. The basic rectangular MSA was improved by adding a rectangular stub of spiral-shaped and an L-shaped slot to achieve tri-band operation. A neutralization line and quadrangular gap in the center of the ground were incorporated to improve isolation by over −27 dB. A MIMO performance parameter ECC of 0.35, a peak gain of more than 1.26 dBi, and overall efficiency better than 76% across all bands were achieved. This study [55] described a reconfigurable double band MIMO MSA for the sub-6-GHz band at resonant frequency 3.5 GHz and ISM band 5.2 GHz. The use of PGP-DGS (Partial Ground and defective Ground structure) and pin diode enabled dual-band operation, while the electromagnetic bandgap (EBG) structure helped to reduce mutual coupling by more than 25 dB and improve isolation where an effective gain of 2.5 dBi was reported.

Complimentary split ring resonator (CSRR)-based compact four port MIMO MSA in [56] for wireless LAN applications resonating at frequencies 2.4 GHz, 2.9 GHz, and 5.8 GHz were investigated. The CSRR structure enabled enhanced antenna bandwidth, compactness, and isolation in the MIMO configuration. Additionally, the peak gain of 4.32 dBi was achieved with low ECC 0.01, a directive gain of 9.98 dB for all operating bands. The 4-element MIMO antenna [57] operated at dual bands, suitable for WiMAX and Wireless-LAN applications. The antenna covered the frequency bands with 400 MHz (3.35–3.75 GHz) and 450 MHz (5.6–6.05 GHz) impedance bandwidths. The isolation between the antenna elements was enhanced by incorporating a parasitic decoupling structure comprising thin L and T-shaped strips. The antenna had a gain of 4.18 dBi at the lower band and 3.62 dBi at the higher frequency band. The antenna exhibited excellent diversity performance with an ECC of 0.01, DG exceeding 9.93 dB, low CCL of 0.4 b/s/Hz, and TARC of −10 dB. The paper [58] described a quad-port double band (27.50–28.35 GHz and 37–37.6 GHz) MIMO MSA operating at 28 GHz and 38 GHz frequencies. The antenna utilized modified circular and semi-circular-shaped slots on radiating patches and DGS (defective ground structure) to improve gain, IBW, and impedance matching. The antenna’s peak gain of 7.9 dB and 13.7 dB was achieved at frequencies of 28 GHz and 38.7 GHz, respectively. The study in [59] offered a quad-port stacked MIMO MSA module with a probe-fed method. The stacked MPA design of the MSA enabled it to cover three different frequencies, at 2.9, 5, and 5.9 GHz, thereby achieving multi-band performance. MIMO MSA used an isolator structure between the patch resulting in a low coupling of less than −23 dB. The antenna exhibited diversity parameters such as ECC less than 0.01, a directive gain of 9.99 dB, radiation efficiency greater than 70%, and a gain 6.4 dBi. The paper [60] presented a novel-shaped, miniaturized quad-band dual-port MIMO antenna that operates well at four different frequencies in a sub-6 GHz configuration, namely 900 MHz, 1800 MHz, 3.5 GHz, and 5.5 GHz. Its C-shaped radiator, with a circular slot in the ground structure, enabled it to have wide bandwidth at some of the bands. The observed findings validated 10 dB impedance bandwidths of (0.72–1.1 GHz) 380 MHz, (1.57–1.90 GHz) 330 MHz, (2.19–4.90 GHz) 2.71 GHz, and (5.30–6.70 GHz) 1.4 GHz, respectively.

The study [61] presented a dual-port MIMO antenna that features a meandering branch as a decoupling structure. The authors proposed two different ground planes, with different shapes where ground-2 is a modification of ground-1 and features a meandering ground branch that acts as a neutralization line and includes a cross-shaped slot to improve antenna port isolation without affecting the antenna performance. The MIMO antenna covers four frequency bands: 0.67–7.29 GHz, 8.07–12.11 GHz, 14.07–15.41 GHz, and 16.04–22 GHz. Furthermore, the antenna exhibits a CCL of less than 0.4 bits/s/Hz, a DG of 10 dB, and low ECC values (<0.008) at its operating frequencies. The study [62] introduced a dual-port MIMO that can radiate in sub-6GHz and millimeter-wave bands, which is suitable for 5G communication systems. The antenna had a unique design with four equally spaced pentagonal concentric slots. The antenna design featured a microstrip line for exciting the sub-6GHz frequency band, and a 1 × 8 power divider coupled through a T-junction arrangement for exciting the millimeter-wave frequency band at 28 GHz with a bandwidth of 0.5 GHz. The sub-6 GHz antenna covered multiple frequency ranges, including 4–4.5 GHz, 3.1–3.8 GHz, 2.48–2.9 GHz, 1.82–2.14 GHz, and 1.4–1.58 GHz. The antenna demonstrated a high radiation efficiency of 91%, a peak gain of 8.5 dBi, and an envelope correlation coefficient (ECC) value of 0.113 at maximum.

In this study [63], a quadband multi-slotted, two-port MIMO was devised for 4G and 5G applications. To reduce mutual coupling at operational frequencies, an I-shaped decoupler structure was incorporated into the ground plane. The antenna exhibited a low ECC of 0.05 and a DG of 9.98 dBi. At 2.5, 3.7, 4.3, and 5.5 GHz, the antenna recorded gains of 3.49, 2.97, 2.93, and 2.54 dBi, respectively, with a minimum efficiency of 79.5% across the bands. In the paper [64], a MIMO antenna was constructed with a modified rectangular patch on the top layer and two rectangular symmetrical slots on the ground structure, enabling dual-band ranging from 5.19–5.41 GHz and 7.30–7.66 GHz. The experimental results demonstrate that the antenna was suitable for Wireless-LAN and X-Band satellite applications. By using orthogonal polarization, the antenna exhibited isolation of 19 dB at both frequency bands. Moreover, the MIMO antenna had a low ECC value of 0.13 and high efficiency of 79.8%. This study [65] presented a reconfigurable multiband MIMO antenna for wireless local area networks. The antenna had two PIFA components coupled with two PIN diodes, and it operated at 2.4, 5.2, and 5.8 GHz when the diodes were on, and at 5.2 and 5.8 GHz when the diodes were off. The frequency ratio was 2.41 and 1.11, respectively. Effective isolation between elements was achieved through the meander line resonator and T slot. The comparison of reconfigurable/multiband MIMO antennas for their performance characteristics is given in Table 3.

### 5.3. Circular Polarized Wideband, Multiband, and Reconfigurable MIMO Antenna for Sub-6 GHz and mmWave Band

**Figure 13:**
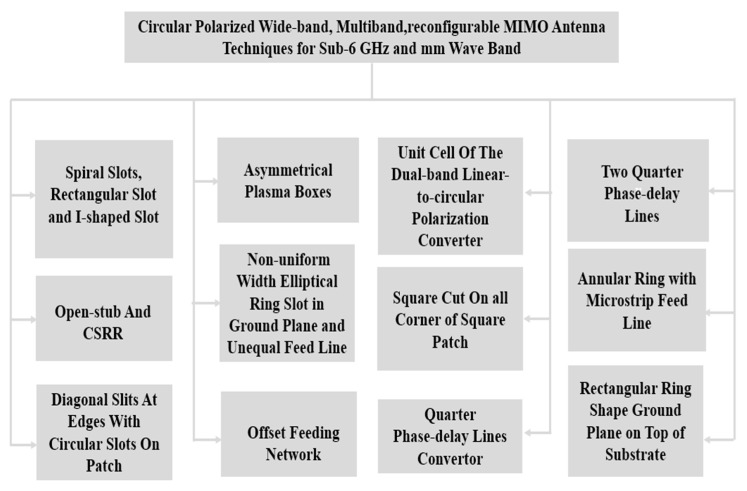
Circular polarized wideband, multi-band, reconfigurable MIMO antenna design approaches.

Linearly polarized antennas used in line-of-sight communication can receive signals with varying power levels. The antenna that receives the weakest signal sets the upper limit for diversity gain and subsequently affects the signal-to-noise ratio (SNR). Additionally, polarization mismatch can lead to insufficient signal gains for highly isolated antennas in both indoor and outdoor environments. However, in recent years, there has been significant research and industrial progress highlighting the importance of circularly polarized (CP) antennas in wireless applications. CP antennas can evenly distribute signal powers among receiving antennas, effectively addressing the issue of polarization mismatch. CP-MIMO (multiple-input multiple-output) antennas improve data rates, capacity, and diversity gain. Some of the circular polarization MIMO antennae with different techniques are listed below in Figure 13.

The study in [66] presented a circularly polarized (CP) two-element MIMO slot-based antenna for UWB applications. The MSA used slots of a spiral, a rectangular, and a stub of L-shaped on the ground to provide broadband circular polarization. Two separate slots were integrated between the mirrored CP square slot antenna components to realize isolation. The MIMO had a 3 dB axial ratio bandwidth (ARBW) of approximately 58% with an effective wideband impedance of 3.17 to 17.39 GHz, and isolation of 16 dB in the entire operational bandwidth. The MSA provided efficient transmission with a peak gain of 4.41 dBi and an efficiency of 70%. The manuscript [67] introduced a circularly polarized textile antenna designed for C-band and future 5G communication, capable of radiating in dual bands (4–18 GHz and 24–58 GHz) and acting as a notch filter for the frequency range 8 to 12 GHz. The antenna demonstrated a bandwidth of 4 GHz and over 34 GHz, and a bending study was performed with positive outcomes. This paper [68] presented an eight-port MIMO antenna that used a CSSR and a stub in the ground for the dual band with elements at the corner to produce CP. The MSA had an operational IBW of 5.75 to 5.95 GHz, and 3.4 to 3.8 GHz and exhibited high isolation between its elements more than 13.8 dB and a low ECC of 0.025. The study presented a double-band circular polarized MIMO antenna [69] at mmWave frequency 28/38 GHz, which achieved left-hand circular polarization on both frequency bands. The design included four slits at the corner, a circular via at the middle of the radiating patch, with shorting pins on the patch’s edges to achieve desired resonance and circular polarization. The design exhibited an FBW of 2.5% at 28 GHz and 32.4% at 38 GHz. Further, the design was transformed into a quad-port MIMO with a coupling among ports reduced to 36 dB. The study in [70] presented a circularly polarized (CP) configuration with a slot incorporated in the ground and coplanar waveguide feeding methods utilized for FR-I NR (New Radio). The asymmetrical coplanar ground structure produced circular polarization, with an effective impedance bandwidth of 2.45 GHz to 8 GHz and an axial ratio (AR) of approximately 82% of the total bandwidth which spans over 2.5 GHz to 6 GHz frequency range. The antenna achieved a gain of 4 dBi, working in both directions and being stable across the entire operating band. The paper presented a small-sized, WB (wide-band), CP dual-port MIMO antenna [71] for FR-I NR. The antenna had 100% 3 dB ARBW from 3.3 to 4.25 GHz with left-handed circular polarization (LHCP) for the whole band. The use of a rectangular ring-shaped ground plane around the patch enabled circular polarization for the entire band. The design achieved an ECC below 0.10 and isolation greater than 15 dB. The manuscript [72] presented a reconfigurable square patch radiator that used plasma polarization for 4G/5G wireless communication. The MSA incorporated two pairs of square boxes of different sizes with orientation angles of 45° and 135° etched with plasma states that varied the polarization. The antenna achieved an isolation of less than 22.46 dB, a gain of 5.8 dBi at a resonance of 2.4 GHz, an ECC of 0.015, and a DG (diversity gain) of 9.95 dB for desired band. The article [73] presented an antenna with orthogonal circular polarization for bands 3.4 to 3.8 GHz which comes under FR-I NR. The design used an elliptical ring slot of non-uniform width etched on the ground and an unbalanced feed to achieve circular polarization. The quad-port MIMO exhibited good performance with an ECC of 0.015 and a CCL of 0.2 bps/Hz. This article [74] presented a wideband triple-polarization MIMO design that utilized slots and shorting pins symmetrically etched on a circular patch characterized by characteristic mode theory. The antenna offered isolation of 35 dB and gain of more than 5.58 dB. A VHF dual-element MIMO antenna with good circularly polarized radiation patterns was described. The antenna offered 24% 3 dB ARBW over 133 to 169 MHz whereas isolation, gain efficiency, an ECC, and directive gain were 25 dB, 3 dBi, 80%, 0.5, and 10 dB, respectively [75]. This paper [76] described a quad-port MIMO antenna that operated in the K/Ka-band for FSS (Fixed Satellite Service). The MIMO antenna was designed using a series of two dipoles of varying lengths that resonated at frequencies between 20 and 30 GHz. An array of 7 × 7 unit cell linear to circular polarization converters was used to generate the orthogonal circularly polarized wave. In both frequency bands, 19.65–22.05 GHz and 29.25–30.35 GHz, the CP antenna exhibited an ECC of 0.19. The study in [77] described a circularly polarized planar, compact MIMO antenna with polarization diversity with an IBW of 2.47 to 2.55 GHz. The MSA employed three stubs and an F-shaped structure on a partial ground plane, effectively reducing mutual coupling between the ports. Additionally, circular polarization was achieved through an offset feeding technique. This study [78] described, circularly polarized, a high isolation of 30 dB, wideband square radiator with tapered edges. Circular polarization resulted due to tapered edges as square-shaped patch corners. The CP-MIMO MSA consists of parasitic components made of a periodic square structure that enabled high isolation, an improved reflection coefficient with effective bandwidth of 4.89 to 6.85 GHz and an ARBW of 3.41 to 6.58 GHz. The antenna offered good MIMO diversity performance with a diversity gain of 10 dB, 80% radiation efficiency, and an ECC of 0.001. A circularly polarized rectangular DRA (Dielectric Resonator Antenna) for MIMO in FR-I NR range 3.57–4.48 GHz with axial ratio bandwidth of 28.33% was achieved [79]. The antenna featured a conformal metal strip to achieve circular polarization and an S-shaped structure on the ground plane to improve isolation between the radiators. This article [80] presented a CP MSA for a 28 GHz mm-wave band and described the different orientations of patches to form the MIMO configuration. The maximum effective impedance bandwidth achieved was 25.5 to 30 GHz with a peak gain of 8.71 dBi, ARBW of 3.1 GHz, and radiation efficiency of 99%. The inverted orientation of the antenna provided optimum results. The study [81] described a two-port circularly polarized crossed-dipole MIMO antenna. The antenna consisted of two orthogonal linear dipoles coupled via two quarter-phase delay lines that exhibit circularly polarized radiation. By incorporating a metallic post in the center of the MIMO configuration, it achieved high isolation of 20 dB across the entire operating bandwidth. The article [82] describes a quad-port MIMO DRA (Dielectric Resonator Antenna) that achieved circular polarization using a distorted dielectric resonator geometry with aperture-coupled excitation and defective ground structure to improve port isolation. The antenna achieved 8.5 to 12.5 GHz impedance bandwidth with axial ratio bandwidth of 9.2 to 10.1 GHz with a gain of 6 dBi. The study [83] introduced a quad-port MIMO circularly polarized patch antenna that used characteristic mode analysis (CMA) and annular ring microstrip feeding to achieve broadband circular polarization. The antenna had isolation greater than 21 dB among ports without decoupling structure. The antenna had effective impedance bandwidth from 5.37 GHz to 11 GHz and an ARBW of 4.65 GHz with a measured gain of 5.69 dBi. The work in [84] presented a small dual circularly polarized (RHCP and LHCP) planar MIMO antenna with effective impedance bandwidth of 3.4 to 3.8 GHz and 100% axial ratio bandwidth for frequencies FR-I NR. For circular polarization, an elliptical-shaped radiator with a horizontal stub was optimized. The antenna for polarization diversity comprised of 4-resonating elements arranged in a mirrored configuration. The optimum separation between elements reduced coupling without a decoupling mechanism. The antenna prototype achieved a maximum gain of 5 dBi, 90% efficiency, an ECC less than 0.1, and 18 dB isolation improvement without decoupling. The comparison of circularly polarized MIMO antennas for their performance characteristics is given in Table 4.

## 6. Outcome of the Review Based on Design Approaches and Isolation Techniques

In this section, some of the major issues and techniques of designing MIMO WB/UWB, multiband, and CP antennas are considered and compared according to their performance. This comparison provides a summary of Section 5 in detail, concerning their applications and future scope for improvement. Various design approaches, isolation techniques, and concluding remarks are summarized in Table 5.

## 7. Design Challenges for 5G MIMO Antennas

In MIMO antennas, there are several challenges to design and integrate in the real environment. The major challenge in MIMO is mutual coupling which reduces the performance of the antenna. Closely spaced antennas give rise to electromagnetic coupling among different antenna ports, thus resulting in degraded performance. Apart from the mutual coupling, the size of the MIMO antenna in the perspective of the portable devices is also a concern to take care of carefully. Multiple antennas require more RF chains along with each antenna element, so the size and cost of the antenna are increased. Thus, techniques such as polarization diversity can be effective in such situations which can reduce the size and cost of the system to some extent. Thus, the researcher has to address different diversity in a single MIMO system carefully to achieve optimal performance. Some of the design challenges are discussed below.

### 7.1. Coupling

In MIMO antennas, multiple antennas at the transmitter and receiver sides are closely packed so resulting in mutual couplings among antenna elements. When multiple antennas are placed in close proximity, they tend to couple electromagnetic energy with each other, which can affect the performance of the MIMO antenna. Coupling can affect radiation patterns and degrade the isolation between antenna elements. There are various methods suggested in the literature to reduce mutual coupling and enhance the isolation among different ports. Parasitic elements, neutralization lines, slots/stubs, decoupling structures, defective ground structures, slot etching, metamaterials, unit cells, etc. are the most widely used isolation techniques [85,86,87,88,89,90]. The parasitic element approach is where an isolating element is placed in between antenna elements to reduce the effects of coupling. In [91], authors describe that the correlation effects between ports are reduced by inserting a parasitic structure. The parasitic structure is designed in a way that reduces the coupling effects in all passbands. Neutralization, slots, and stubs are methods utilized on the ground plane or between radiating patches which reduce the effect of coupling with another antenna. In [92,93,94], the papers describe the neutralization techniques along with SIW, slots, or decoupling networks to reduce the isolation. As the literature shows, neutralization line, slots, and stubs are easily integrable with structure and provide significant improvement in isolation among different ports. Moreover, for practical applications, common ground plane is required due to which current in the ground plane excites the other antenna element and degrades the performance of the antenna in terms of radiation pattern and efficiency. Thus, defective ground structures or slots on the ground plane significantly reduce the surface current to excite the other antenna, which in turn provides a greater degree of isolation among ports [95]. Metamaterials structures designed on the radiating element, ground plane, or between antenna elements significantly improve the isolation. Structures such as SRR (split ring resonator), CSSR (complementary split ring resonator), and unit cells most widely used isolation techniques in the literature. In [96,97,98], the authors described an isolation techniques based on the metamaterials and DGS provides significant improvement.

### 7.2. The Compactness of Portable Devices

Meeting the growing need for increased mobile data volume necessitates enhanced capacities in radio mobile networks. While network densification and the integration of new frequencies are potential solutions, incorporating higher-order MIMO capabilities into existing radio networks presents an opportunity to significantly enhance peak data rates and augment overall network capacity. In conventional 2 × 2 MIMO networks, cross-polarized antennas are commonly utilized. To introduce additional, independent antennas into the system (e.g., 4 × 4 MIMO in smartphones), a second cross-polar antenna can be employed [99]. To ensure the antennas are uncorrelated, it is necessary to have either horizontal or vertical spacing between them in a handheld device; it is a challenge to the researcher to design a such configuration. Currently, mobile phones available in the market most of the mid-range smartphones support a 2 × 2 MIMO antenna or a maximum of 4 × 4 MIMO in the premier segment of smartphones. MIMO antennas need to be compact to be integrated into portable devices. However, miniaturizing antennas can compromise their performance. Designers must balance the trade-offs between size, efficiency, and bandwidth.

### 7.3. Polarization Diversity

In practice, large antenna spacings are often required to achieve significant multiplexing or diversity gain. The use of dual-polarized antennas (polarization diversity) is a promising cost- and space-effective alternative, where two spatially separated uni-polarized antennas are replaced by a single antenna structure employing orthogonal polarizations [100]. This can be achieved by using antennas with different polarization orientations or by using dual-polarized antennas. Designers must carefully choose the antenna types and orientations to achieve optimal performance.

### 7.4. Frequency Band Coverage

5G wireless network operates on the frequency bands sub 6 GHz and mmWave for 5G communication. Both Sub 6 and mmWave band have their pros and cons. The mmWave technology provides a higher data rate due to the large bandwidth availability but low coverage area because of more attenuation at high frequencies. The sub-6 GHz band has low attenuation loss with greater coverage area and lower data rate due to the limited bandwidth availability [101]. Thus, it is of utmost necessity to design a MIMO antenna which supports multiband operation for FR-1 and FR-2. Thus, it is necessary to ensure the antenna bandwidth and radiation characteristics are matched to the frequency bands of interest.

## 8. Conclusions

A comprehensive review of MIMO antenna design approaches has been discussed in this paper. WB/UWB characteristics were achieved through different modifications of radiating patch, ground plane and feeding network. H-P shaped, E-L shaped, annular ring, DGS, partial ground plane, and metamaterial structure were employed to achieve wideband or ultra-wideband properties. Multiband MIMO antennas can be achieved through different aspects such as characteristic mode theory, stubs and slots on radiating patch, EBG structure, SRR or CSRR, and reconfigurable patch antenna. Moreover, the CP-MIMO antenna can be designed by optimizing the radiating patch, ground plane, and feed network through different techniques. It was observed that using the spiral slot on radiating patches, circular patches, tapered edges of patches, or diagonal slots on patches leads to circular polarization. Quarter phase delay lines, plasma boxes, circular rings or defects in the ground plane, split ring resonator, or complementary split ring resonator are other promising design techniques to obtain a circularly polarized antenna. Apart from MIMO antenna design techniques for WB/UWB, multiband and CP-MIMO isolation among different ports have been the utmost priority. In this study, different MIMO configurations with decoupling structures were also presented. Some of the decoupling networks or structures are neutralization line, meander line, isolation improvement based on SRR or CSRR structures, shorting pin, parasitic elements, slots with DGS, and feeding network, which are promising techniques to reduce the isolation among the ports, leading to the design of high-performance MIMO antennas. Thus, in this review paper, different design aspects of MIMO antenna for NR FR-1 and FR-2 were studied, which will be helpful for researchers to further fulfil the demand for high data rate in 5G and B5G cellular generation.

## Figures and Tables

**Table 1:** Cellular generation evolution and their characteristics [9].

Cellular Generation	Frequency Bands	Channel Bandwidth	Modulation Waveform	Access Techniques	Peak Data Rates	Spectral Efficiency	Use Cases
2G	900 MHz, 1800 MHz	200 KHz	GMSK	TDMA	114 kbps in the DL and 20 kbps in the UL	-	Voice and messaging services to mobile users.
3G	2100 MHz	5 MHz	OFDM	WCDMA	DL speed- 21 Mbps UL Speed- 5.7 Mbps	<1 bit per second per Hertz (bps/Hz)	Video Conferencing and High-Speed Data Network
4G	700 MHz, 800 MHz, 900 MHz, 1700/2100 MHz, 1800 MHz, 1900 MHz, 2300 MHz, 2500 MHz, and 2600 MHz	20 MHz	Modulation (QAM) and Orthogonal Frequency Division Multiplexing (OFDM)	(OFDMA) as the multiple access technology for downlink transmission, and Single Carrier Frequency Division Multiple Access (SC-FDMA) for uplink transmission	20 MHz channel bandwidth and 4 × 4 MIMO, the theoretical peak data rate is 1 Gbps for downlink and 500 Mbps for uplink	2–4 (bps/Hz)	Higher-speed data services, lower latency, and better quality of service compared to 3G networks video streaming, online gaming
5G	Sub-1 GHz, 1–6 GHz, and above 6 GHz, including millimetre-wave (mm-wave) frequencies	sub-6 GHz- 100 MHz and mmWave- up to 400 MHz	(QPSK), 16 QAM, 64QAM, 256QAM	OFDMA—Primary Multiple Access Technology DL Transmission, SC-FDMA—single-carrier frequency division multiple access—UL Transmission	20 Gbps for downlink (DL) and 10 Gbps for uplink (UL)	Upto 30 bps/Hz	(eMBB), massive machine-type communications (mMTC), and ultra-reliable low-latency communications (URLLC)

**Table 2:** Comparison of wideband MIMO antennas for their performance characteristics.

Ref.	Size of MIMO Antenna (mm^3^)	No. of Antenna Element	Operating Band (GHz)	Impedance Bandwidth (GHz)	Efficiency (%)	Isolation Techniques	ECC	Isolation (dB)	Gain	DG (dB)	CCL (bps/Hz)
[29]	100 × 40 × 1.6	10	24.25–27.5	3.25	77	DGS and DMS	0.1 × 10^−6^	−38	2.2–5.3 dBi	10	0.005
[30]	24 × 24 × 0.254	4	25–39	14	92.5	Tilted patch with extended arm in the middle of MIMO array	<0.05	−26	7.1 dBi	-	-
[31]	150 × 75 × 0.8	8	(−6 dB Bandwidth) 3.3–8.5	5.2	40–75	H-T shaped slot in ground plane	0.007	>−10	6 dBi	7–8.8	-
[32]	95 × 65 × 0.2	8	3.05–3.74	0.69	95	-	<0.01	>−15	2.2 dBi	-	-
[33]	30 × 40 × 1.6	4	3.20–5.85	2.65	85	Un-protruded multi-slot (UPMS) on ground plane	<0.05	−17.5	3.5 dBi	9.98	-
[34]	23.7 × 8.8 × 0.51	4	36.83–40.0	3.17	80	Orthogonal orientation of element for MIMO Array	0.001	−25	6.5 dB	10	0.6
[36]	150 × 75 × 7 (Smartphone Model) Ant. Pair 30 × 7	4	(−6 dB Bandwidth) 3.3–7.5	4.2	78	Decoupling capacitance in ground plane	0.06	>−10	-	-	-
[38]	45 × 66 × 0.625	4	2.21–6	3.79	41	-	0.016	>−15	0.53 dBi	9.9–10	-
[39]	48 ×35 ×1.6	2	2–18	16	85%	Metamaterial (SRR) and DGS	<0.07	−27	2–8 dB	9	-
[40]	146 × 146 × 1.524	4	3.27–3.82	0.55	92%	Slots and shorting pin	<0.001	>−32	8.1 dBi	9.99	-
[45]	150 × 75 × 7.8	8	(−6 dB BW) 3.2–6	2.8	38% to 83%	No coupling techniques	<0.31	−12.6	-	10	-
[46]	150 × 71× 1.6 (Smartphone Model) Ant. Element 30 × 7 × 1.6	8	(−6 dB BW) 3.3–5.0	1.7	63 to 86%	Self-Decoupled	<0.065	>−11.5	-	-	-
[49]	24 × 22.5 × 1.6	4	26–40	14	70%	Orthogonal orientation of antenna	<0.02	>−20	>10.2 dBi	>9.9	-
[50]	24 × 24 × 0.835	4	24.8–44.45	19.65	>85%	Orthogonal orientation of antenna	<0.007	>−20	8.6 dBi	9.96	-
[52]	36 × 38× 1.6	2	2.1–16.9	14.1	-	Partial ground, Slots/Stubs on ground plane	0.018	>−16	4.5 dB	>9.997	-

**Table 3:** Comparisons of reconfigurable/multiband MIMO antenna characteristics.

Ref.	Size of MIMO Antenna (mm^3^)	No. of Antenna Element	Operating Band (GHz)	Impedance Bandwidth (MHZ/GHz)	Efficiency (%)	Isolation Techniques	ECC	Isolation (dB)	Gain	DG (dB)	CCL (bps/Hz)
[53]	80 × 54 × 1.6	2	1.06–1.24, 1.52–2.13, 2.19–2.63	180 MHz, 610 MHz, 440 MHz	-	Mushroom-Inspired EBG Structure with 180° out of phase SISO Elements	0.5	−15	3.8 dBi	9.5	-
[54]	32 × 26 × 1.5	2	2.4–2.5, 3.3–3.6, 4.8–5.0	100 MHz 300 MHz 200 MHz	76	Neutralization Line and DGS	0.35	<−27	1.26 dBi	-	-
[55]	26 × 31 × 1.6	2	2.8–3.6 4.7–5.6	800 MHz, 900 MHz	-	dual-band EBG	-	<-25	2.5 dBi	-	-
[56]	106.6 × 106.6 mm^2^	4	2.29–2.49, 2.85–3.04, 5.61–5.80	200 MHz 190 MHz 190 MHz	-	CSRR	9.92 × 10^−4^, 4.50 × 10^−4^, 6.00 × 10^−6^	>−20 >−20 >−24	3.94 dB 3.80 dB 4.32 dB	9.98	-
[57]	34 × 34 ×1.6	4	3.35–3.75 5.6–6.05	400 MHz 450 MHz	~87	Parasitic Metal Strips	<0.01	−19(min)–48(max)	4.18 dB 3.62 dB	>9.93	-
[58]	43.611 × 43.611 × 0.4	4	27.50–28.35 37–37.6	850MHz 600MHz	98 (28 GHz) and 91.7 (37.3 GHz)	-	<2.5 × 10^−4^ <3.5 × 10^−4^	>−20 >−30	7.9 dB 13.7 dB	10	-
[59]	65 × 65 × 1.6	4	2.92–2.97, 4.98–5.22, 5.78–6.02	50 MHz 240 MHz 240 MHz	>70	Isolator structure	<0.01	>−23	6.4 dBi	~9.99	0.05 (first band) <0.01 (other bands)
[60]	42 × 40 × 0.02	2	0.72–1.1 1.57–1.90 2.19–4.90 5.30–6.70	380 MHz 330 MHz 2.71 GHz 1.4 GHz	85	Diagonal Rectangular Strip within the Circular DGS	≤0.02	−22 to −36	-	~10	0.04 0.04 0.01 0.03
[61]	30 × 20 × 1.6	2	0.67–7.29, 8.07–12.11, 14.07–15.41, 16.04–22	6.62 GHz 4.04 GHz 1.34 GHz 5.96 GHz	70-93	Neutralization Line and Slots	<0.008	>−18	3.37–6.2 dB	10	<0.04
[62]	60 × 120 × 0.508	2	4–4.5 3.1–3.8, 2.48–2.9, 1.82–2.14, 1.4–1.58 27.8–28.3	500 MHz 700 MHz 420 MHz 320 MHz 180 MHz 500 MHz	76–91	DGS	0.091–0.113	−14 dB	2.2–8.5 dB	9.936–9.999	-
[63]	18.5 × 56 × 1.6	2	2.43–2.57 3.37–3.80 4.25–4.5 5.09–6.25	140 MHz 430 MHz 250 MHz 1.16 GHz	>79.5	Decoupling Strips at Ground Plane	0.05	−22.44 to −28.98	2.54–3.49 dBi	9.98	0.4
[64]	1.6 × 27 × 21	2	5.19–5.41 7.30–7.66	220 MHz 360 MHz	>79.8	Orthogonal Polarization of MIMO Elements	0.13	>−19	9.38 dB	9.78	-
[65]	77 × 52× 1.6	2	2.37–2.48 4.8–5.9	110 MHz 1100 MHz	-	Meander line resonator and T slot	-	>−20	2.31–3.75 dB	-	-

**Table 4:** Comparisons of circular polarized reconfigurable/multiband/wideband MIMO antenna characteristics.

Ref.	Size of MIMO Antenna (mm^3^)	No. of Antenna Element	Operating Band (GHz)	ARBW (GHz)	Efficiency(%)	Isolation Techniques	ECC	Isolation (dB)	Gain	DG (dB)	CCL (bps/Hz)
[66]	40 × 20 × 1.6	2	3.17–17.39	4.8–8.8	70	-	0.005	−16	4.416 dBi	9.98	0.26
[68]	174 × 85 × 0.8	8	3.4–3.8, 5.75–5.95	5.75–5.95	-	-	0.025	>−13.8	4.2 dB	-	-
[69]	75 × 100 × 0.508	4	27.6–28.3 35.2–47.2	27.6–28.3 35.2–47.2	96.7 and 96.41	DGS	~0.005	−36	7.03 dB 7.368 dB	>9.985	-
[71]	37 × 30 × 0.8	2	3.2–4.25	3.2–4.25	>95	-	<0.10	−15	1–3 dBi	9.94	-
[72]	160 × 160 × 4.6	4	2.38–2.53	2.42–2.46	-	-	0.015.	>−22.44	5.8 dBi	9.95	-
[75]	89 × 80 × 1.57	2	0.133–0.169	0.133–0.169	80	-	<0.5	>−25 dB	~3 dBi	~10 dB	-
[77]	150 × 100 ×0.8	2	2.47–2.55	2.50–2.66	56.5 to 91.6	DGS and rounded stubs	0.003	−20 dB	4.49 dBi to 6.07 dBi	-	-
[78]	70 × 40 × 1.6	2	4.89−6.85	5.41–6.57	80	Square parasitic elements	0.001	−30	6.45 dB	~10	<0.4
[80]	20.5 × 12 × 0.79	4	25.5–30	25.2–28.3	99	-	-	−35	8.71 dB	-	-
[81]	150 × 75 × 1.6 (Smartphone Model)	4	3.46–3.86	3.58–3.73	-	-	0.02	−20		-	-
[82]	35 × 30	4	8.5–12.5 GHz	9.2–10.1	80	DGS	<0.005	>−22	6 dB	>9.96	<0.4
[83]	50 × 50 × 0.4	4	5.37–11	5.61–10.26	-	-	0.003	−21	5.6 dB	~10	-
[84]	40 × 40 × 1.6	4	3.4 to 3.8	3.4 to 3.8	90	-	0.1	−18	5 dBi	-	-

**Table 5:** Design approaches, isolation techniques, and remarks for 5G MIMO antennas.

S. No.	Design Approaches/Isolation Techniques for WB/UWB, Multiband, and CP MIMO Antenna	Concluding Remarks
1	Defected ground structure (DGS) and defected microstrip structure (DMS) with stub-loaded co-planner waveguide (CPW) [29].	DGS and DMS loaded structures with CPW ground plane improve the isolation as well as bandwidth and defected metallic plane back side of the substrate improves the co-polarization to cross-polarization ratio. DGS-DMS structure significantly improves the isolation below −30 dB, also, co-polarization to cross-polarization shows excellent result.
2	Stub-loaded, partial ground plane and DGS structure with I-shaped resonator and orthogonal orientation of antenna with line resonator [30].	Stub, DGS, and PGP effectively increase the bandwidth. Moreover, the stub provides impedance matching and improves the radiation characteristics of the antenna. The neutralization line along with the orthogonal orientation of antennas in MIMO configuration and neutralization lines, this hybrid approach greatly reduces mutual coupling.
3	PGP, inverted L-shaped slot with a circular ring on the corner of the patch. Edge-to-edge spacing of the antenna in the horizontal and vertical planes for isolation [32].	Again, a partial ground plane with a slot structure is responsible for the wide impedance bandwidth. Edge-to-edge placement for an antenna in MIMO provides isolation, but not to that significant a level, but also increases the size of the antenna to some extent.
4	Monopole antenna with PGP, E-L slots, and stubs. Novel unperturbed multi-slot (UPMS) on the ground plane with PGP as an isolation technique [33].	Partial ground plane, slots on the patch, and stub to improve the antenna’s radiation characteristics as well as impedance bandwidth, whereas UPMS structure on connected ground plane enhances the isolation to some extent but minimum isolation is still −20 dB and more improvement is needed.
5	Modified rectangular patch to HP-shaped configuration with the asymmetrical orthogonal orientation of antenna for isolation techniques [34].	Modified rectangular patch configuration shows significant improvement in the impedance bandwidth due to additional resonances. Moreover, this kind of placement of the antenna shows greater isolation improvement with minimum isolation is −25 dB and maximum isolation achieved up to −45 dB. However, the limitation of such a configuration is the size of the antenna is compromised.
6	PGP with a circular resonator and a circular stub connected via a tilted line for wideband impedance and antenna are placed edge to edge vertically and horizontally to avoid mutual coupling [38].	This configuration provides a wide impedance due to the partial ground plane and circular patch and stub approach. However, due to no decoupling structure being utilized for improvement of isolation, mutual coupling for the connected ground case is a minimum of −15 dB. Thus, edge-to-edge spacing between MIMO antennas is a simple approach to designing the antenna, but isolation is compromised. If the spacing between antenna elements is increased, it will improve the isolation to a great extent but it increases the size of the antenna.
7	Metamaterial-based PGP, DGS, and modified rectangular patch using slots and tapered edges. SRR (split ring resonator) structure between two patches for isolation improvement [39].	Application of Metamaterial SRR structure along with PGP, DGS, and modified patch antenna significantly improves the isolation gain along with impedance bandwidth. A good isolation below −20 dB is achieved in the entire band. Moreover, the efficiency of the antenna improved with the application of the SRR structure.
8	Rectangular patch with lower ends truncated via semi-circle and metasurface on the top of substrate and patch at top of the lower substrate with ground printed on the back [40].	Metasurface as superstrate with patch antenna significantly improves the impedance bandwidth, peak gain, and efficiency of the antenna. Metasurface designed properly generates additional resonances which effectively increase the bandwidth and gain of the antenna. The limitation to using such a configuration height of the antenna is increased.
9	Metamaterial-based CMSPR (combined metamaterial structure and parasitic ring) unit cells triple line MIMO dipole antenna. Metamaterial as a superstrate is on the top of the antenna in the horizontal plane XY and a metamaterial wall in the YZ plane between two antenna elements [41].	A conventional approach to designing printed dipole antenna without metamaterial structure has suffered from low bandwidth and gain. As CMSPR structure is used as a superstrate, it not only increases the impedance bandwidth but improves the gain of the antenna a lot. CMSPR as a wall in between antenna elements provides a significant improvement in the isolation gain with minimum isolation below −20 dB and maximum isolation up to −50 dB. This technique is quite useful for improvement in the gain and impedance bandwidth of the antenna, but design complexity is higher compared to the other design approaches.
10	A novel multiband equilateral triangular patch antenna (ETPA) using PIN diode-based approach with PGP and mushroom-inspired electronic bandgap (EBG) structure for isolation [53].	In this approach, a multiband reconfigurable MIMO antenna is designed using a PIN diode with a small frequency ratio. The 1800 orientation of radiating element with mushroom-inspired EBG structure provides isolation between the element and below −15 dB in the entire band. Moreover, the advantage of using an EBG structure is that edge-to-edge spacing between elements can achieve λ_l/20 where λ_l is the wavelength at a lower frequency band. Thus, the MIMO antenna can be compact in nature.
11	Modified conventional rectangular patch using spiral-shaped stub on the top and L-shaped slot for multiband using characteristic mode analysis (CMA) and MLS (meander line and a split ring) technique for isolation improvement [54].	CMA results in the rectangular patch antenna determining the stub and slot position on the patch. Further using a PIN diode reconfigurable antenna is presented. Using the MLS structure between two radiating patches and a slot on the ground plane combinedly improves the isolation of more than −18 dB across all bands. The theory of characteristic modes (TCM) study is used to explain the behavior of antenna and design modification based on the modal current distribution and mode coupling on the radiating element.
12	CSRR-based multiband rectangular patch antenna [56].	CSRR structure on the ground plane results in high isolation among ports below −20 dB along with edge-to-edge to the spacing between elements is λ_l/4 at the lowest frequency band. Moreover, CSRR is responsible for additional resonant modes that result in the antenna to multiband operation. This is a simple technique to achieve multiband, but isolation can improve more.
13	An open-ended L-slot on the curved monopole for multiband with parasitic elements, inverted L-shaped thin metal strips, and orthogonally placed elements for isolation [57].	The slot method and curved radiating element for multiband and parasitic elements along with an inverted L-shaped thin metallic line on the ground plan improve the isolation. The advantage of the parasitic element is easy to integrate with the MIMO antenna and shows a significant improvement in isolation gain.
14	Cross-shaped DGS with combinations of circular and semi-circular-shaped slots for radiating elements for multiband and isolation [58].	Circular and semi-circular slots on the patch for dual-band operation and DGS with circular slot reduces the coupling between ports. This technique provides excellent isolation.
15	Modified I-shaped radiator with CPW feed incorporating the two spiral slots on the opposite corner of the radiating element for circular polarization (CP) [66].	This design approach provides the bidirectional characteristics of the CP antenna with RHCP and LHCP in +z and −z, respectively, due to the interchange of the CPW ground plane and spiral slots on the corner. Minimum isolation was achieved below −16 dB with 58% axial ratio bandwidth. The advantage of the design is that easy to get CP with a reasonable axial ratio bandwidth (ARBW).
16	Diagonal slits at the corner of the rectangular patch antenna and edge-to-edge spacing for isolation [69].	A more conventional way to achieve CP is to cut diagonal slits at corners with holes at the center of the patch.
17	The asymmetric ground plane in CPW configuration and wide slot on the back with stub results in a CP antenna [70].	Due to asymmetric CPW, ground plane current distribution on the monopole and CPW ground plane generates the horizontal and vertical current components which are mutually perpendicular results in the CP mode generated. The advantage of this approach is that a good axial ratio is obtained and it is easy to design. In addition, the design is bidirectional with RHCP and LHCP by interchanging the positions of the CPW ground plane along with back slots and stubs providing polarization diversity.
18	T-shaped radiators with ring shape rectangular ground plane, both on top of the substrate [71].	This approach generates the LHCP due to the clockwise distribution of the current on the radiator and ground plane. The simplest method to achieve CP with a narrow band with 100% axial ARBW can be obtained.
19	Two feed line systems branching from the main feed as C and I shaped feed with an asymmetric elliptical slot on the ground plane result in CP [73].	This approach can be used for polarization diversity and due to mirroring each other, polarization senses are opposite to each other, with slots on the ground plane improve the isolation among ports.
20	Offset feeding with mirrored F-shaped stubs for isolation [77].	Offset feeding to a rectangular patch antenna is the simplest technique for CP. DGS improves the isolation, and impedance matching. Furthermore, the mirrored orientation of patches for MIMO provides bidirectional CP which results in good polarization diversity.
21	Corner-cut square patch with probe feed and parasitic elements for isolation and improvements in impedance bandwidth [78].	Square cut on all sides of the patch with probe feed results in CP with parasitic element around the antenna providing better isolation. The advantage of this technique is that it is easy to design and good ARBW is achieved.

## Data Availability

Not applicable.
